# Predictive factors for relapse of cryptogenic organizing pneumonia

**DOI:** 10.1186/s12890-018-0764-8

**Published:** 2019-01-09

**Authors:** Zenya Saito, Yugo Kaneko, Tsukasa Hasegawa, Masahiro Yoshida, Kyuto Odashima, Tsugumi Horikiri, Akira Kinoshita, Keisuke Saitoh, Kazuyoshi Kuwano

**Affiliations:** 1 Division of Respiratory Diseases, Department of Internal Medicine, The Jikei University Daisan Hospital, 4-11-1 Izumihoncho, Komae-shi, Tokyo, 201-8601 Japan; 2 Division of Respiratory Diseases, Department of Internal Medicine, Atsugi City Hospital, Kanagawa, Japan; 3 Division of Respiratory Diseases, Department of Internal Medicine, The Jikei University School of Medicine, Tokyo, Japan

**Keywords:** COP, Predictive factor, Relapse, Steroid, Radiographic findings

## Abstract

**Background:**

Relapse of cryptogenic organizing pneumonia (COP) may lead to poor long-term prognosis and necessitates multiple rounds of steroid treatment with potential adverse effects. The objective of this study is to identify predictive factors of COP relapse by comparing demographic and clinical variables between relapse and non-relapse groups.

**Methods:**

During 2008–2013, 33 COP patients were treated, of which 23 (69.7%) and 10 patients (30.3%) were assigned to the non-relapse and relapse group, respectively. From medical records, we compared the following variables at initial episode: clinical characteristics, serum parameters, chest CT scan findings, and steroid treatment.

**Results:**

Clinical characteristics, cumulative prednisone dose, and steroid treatment duration were similar between groups. In univariate analysis, alternatively, the proportion of patients with bilateral shadow pattern, traction bronchiectasis, and partial remission after steroid treatment was significantly higher in the relapse group. These differences were not significant by multivariate Cox regression analysis.

**Conclusions:**

We identified radiographic findings, such as bilateral shadow pattern, traction bronchiectasis, and partial remission, may have possibility of predictive factors for COP relapse. Larger-scale studies are required to confirm if any are independent predictors of COP relapse.

## Background

Organizing pneumonia (OP) is pathologically defined by the presence of granulation tissue buds within distal pulmonary airspaces consisting of fibroblasts and myofibroblasts intermixed with loose connective matrix [1]. Clinically, OP is associated with a syndrome of acute or subacute onset that may include cough, fever, and progressive dyspnea, and radiographically, with multiple patchy alveolar opacities not responsive to antibiotics.

Organizing pneumonia is classified as either cryptogenic OP (COP), which has no specific etiology, or secondary OP caused by an inflammatory reaction to drugs, infection, collagen vascular disease, malignancy, or radiation therapy [2–4]. One previous study [5] reported that secondary OP was less responsive to treatment and associated with worse prognosis than COP, while another [6] reported similar clinical and radiographic findings, treatment responses, relapse rates, and mortality for COP and secondary OP. Patients with OP sometimes relapse during or after steroid treatment, and several relapses increase the side effects of steroid treatment and may negatively influence prognosis. The frequency of relapse ranges from 9 to 33% [7–9], and we sometimes encounter relapse when steroids are tapered or stopped. Therefore, it is important to identify predictive factors of relapse as possible aids to guide preventative treatment.

A relationship between relapse and steroid dose was identified [10, 11], and guidelines for steroid treatment have been proposed by Epler [3] and Schwartz and King [4]. Epler suggested starting with 1 mg/kg/day prednisone (60 mg/day) for 1–3 months, tapered to 40 mg/day for 3 months, and then 10–20 mg/day for 1 year. Schwartz and King suggested initiating therapy with 1–1.5 mg/kg/day prednisone for 4–8 weeks, tapering to 0.5–1 mg/kg/day for the ensuing 4–6 weeks. Although there is general consensus on steroid efficacy, the specifics of the steroid treatment regimen, such as initial dose and interval of tapering, have not been standardized. Several studies reported that relapse was associated with tapering or discontinue of steroid treatment [6, 12]. Other proposed predictive factors for relapse are severity of initial hypoxemia, traction bronchiectasis and architectural distortion on computed tomography (CT) scans with increased serum Krebs von den Lungen-6 (KL-6) levels, the presence of intra-alveolar fibrin in lung biopsy tissue, and the delay between first symptom and treatment onset [10, 12–15]. However, the predictive factors for relapse remain uncertain.

The purpose of this study is to identify predictive factors for relapse of COP by comparing the clinical characteristics, laboratory data, radiographic findings, duration of steroid treatment, cumulative equivalent dose of prednisone, and response to steroid therapy between non-relapse and relapse groups.

## Methods

### Study population and selection criteria

We retrospectively reviewed the medical record of patients admitted to the Jikei University Daisan Hospital between April 2008 and March 2013. The following criteria were required for inclusion in the present study: (1) histopathological reports based on transbronchial lung biopsy expressly describing the presence of granulation tissue buds in the distal airspaces, (2) clinical and imaging features compatible with COP such as fever, cough, dyspnea, and good response to steroid treatment, and (3) steroid treatment for COP. Patients were excluded for the following criteria: (1) secondary OP induced by infections, drugs, or collagen vascular diseases, (2) spontaneous improvement without treatment, or (3) malignant tumors. Patients were classified as relapse or non-relapse based on standard criteria (below). This study was approved by the institutional research ethics committee of the Jikei University School of Medicine, and written informed consent was obtained from all patients. All patient records were anonymized prior to analysis.

### Definition of relapse

**Fig. 1:**
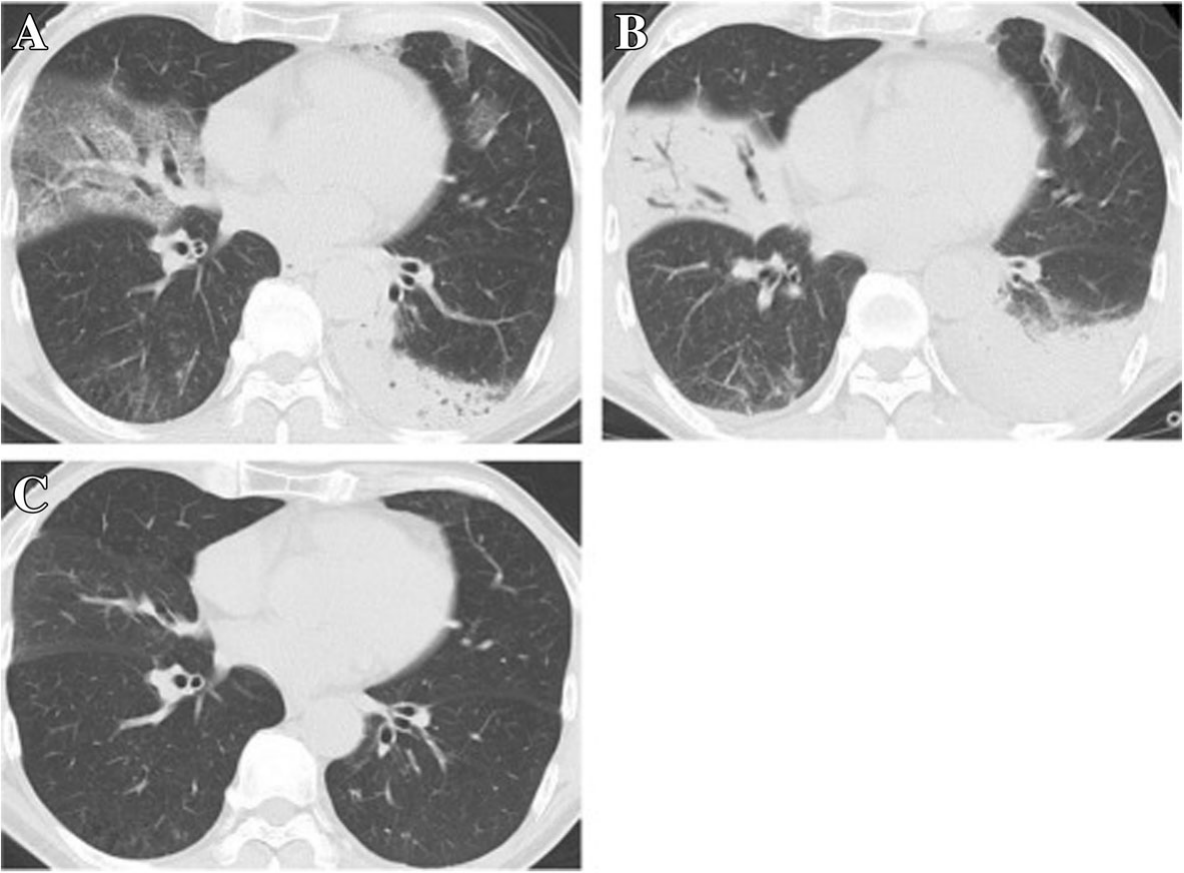
Typical CT images of non-relapse case. **a** Ground-glass attenuation with traction bronchiectasis in the right middle lobe and consolidation in the left lower lobe were recognized at the first visit. **b** Ground-glass attenuation changed into consolidation in the right middle lobe and consolidation apparently expanded in the left lower lobe just before treatment. **c** All shadows completely vanished after three months and relapse didn’t occur after one year

**Fig. 2:**
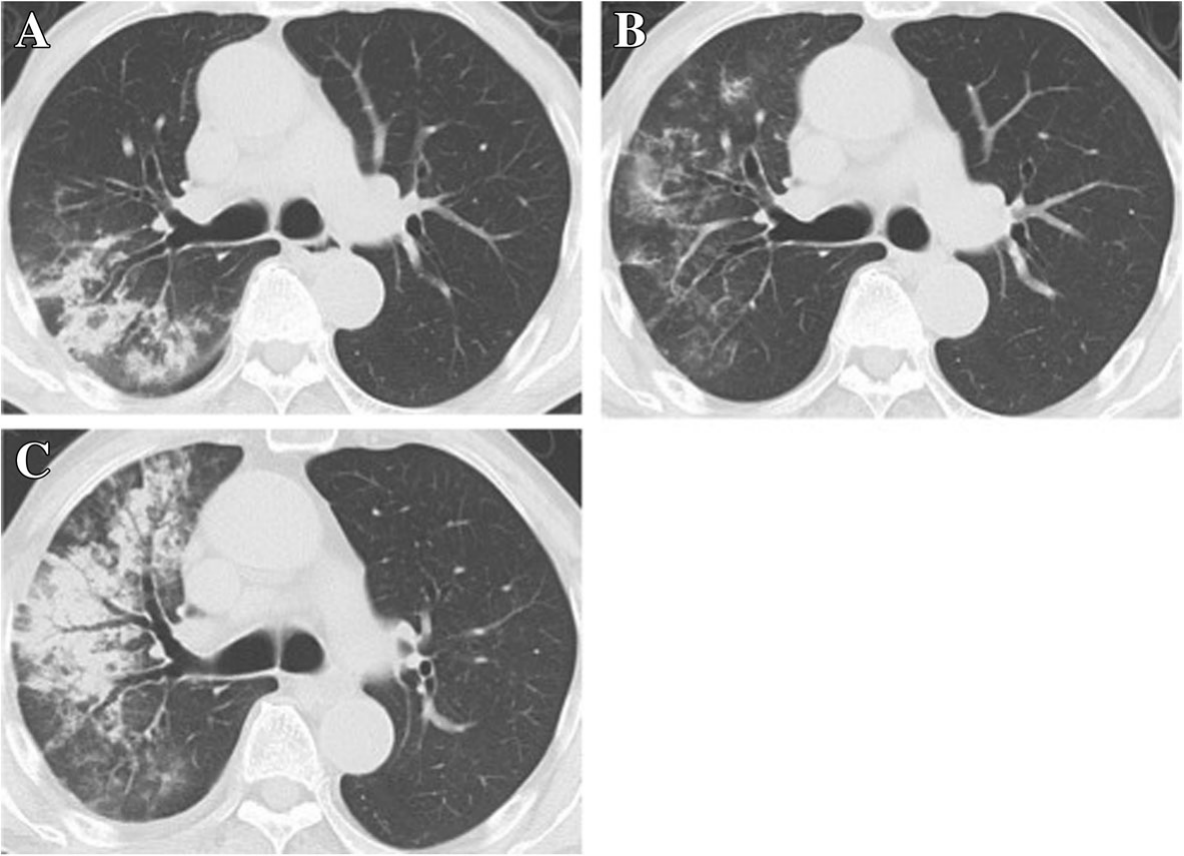
Typical CT images of relapse case. **a** Consolidation and nodules in the right upper lobe were recognized at the first visit. **b** These shadows remained without vanishing completely after treatment. **c** Relapse occurred after six months of treatment

Relapse was defined as follows: (1) the appearance of characteristic new infiltrates and/or the exacerbation of residual target shadow on chest CT scans as well as COP-compatible clinical features during or after steroid treatment, (2) the improvement by increasing steroid treatment only without antibiotics. Cases conforming to this definition did not require histopathologic proof of COP for the diagnosis of relapse. Typical images of non-relapse and relapse case were presented in Figs. 1 and 2.

### Data analysis

We retrospectively compared the following variables from medical records at initial episode between relapse and non-relapse patients: sex, age, smoking status, symptoms and signs, white blood cell (WBC) count, lactate dehydrogenase (LDH), alkaline phosphatase (ALP), C reactive protein (CRP), KL-6, surfactant protein D (SP-D), chest CT scan findings (pattern and location of lesion), steroid treatment duration, cumulative equivalent dose of prednisone (mg/kg), reaction to steroid therapy. In reaction to steroid therapy, we compared the proportion of partial remission between groups and defined it as the presence of residual shadow that had not completely disappeared during tapering or after the completion of steroid treatment in CT scans.

### Review of radiology

All patients had chest CT scans. Interpretation reports of all CT scans by radiologists were available for review. The pattern of abnormal findings was classified as consolidation, ground-glass attenuation, or nodules. The images were assessed for the distribution of lung parenchymal findings and the presence of sequelae such as traction bronchiectasis and pleural effusion. The distribution of abnormal findings was classified as unilateral or bilateral and involving the upper, middle, or lower lung zones. Regarding the distribution, the type of shadow wasn’t distinguished. Ultimately all chest CT scans were reviewed and interpreted by two radiologists and three respiratory physicians, and discussions were held on findings if necessary. Finally, findings were identified by agreement of members.

The definitions of radiological terms [16] are as follows: consolidation was defined as areas of increased attenuation obscuring normal lung markings, ground-glass opacity as hazy attenuation without obscuration of the underlying vessels, nodules as coin-like shadows < 3 cm in diameter, and traction bronchiectasis as irregular bronchial dilatation surrounding parenchymal abnormalities caused by repeated cycles of infection and inflammation.

### Review of shadow distribution

We reviewed the shadow distribution from CT scans to investigate the relationship between relapse and shadow distribution. The right lung has three lobes; upper, middle and lower. The left lung has two lobes: upper and lower. Each lobe is supplied by an individual lobar bronchus. Right upper lobe contains 3 segments (apical, posterior, and anterior), right middle lobe 2 (lateral and medial) and right lower lobe 5 (superior, medial, anterior, lateral, and posterior). Left upper lobe contains 4 segments (apicoposterior, anterior, superior lingular, and inferior lingular) and left lower lobe 3 (superior, anteromedial, lateral, and posterior). Based on these anatomical structures, we distributed all shadows on CT images to each lobe.

### Review of treatment

We retrospectively examined the treatment records of all patients. To evaluate the relationship between relapse and steroid treatment, we expressed steroid dose in prednisone equivalents and compared the cumulative dose (in mg/kg) between relapse and non-relapse groups. Additionally, we compared the differences in total steroid treatment period and follow-up period between these groups.

### Statistical analysis

The Mann–Whitney U test was used to compare means of continuous variables and the chi-square test to compare proportions between groups. Multivariate Cox regression analysis was performed to evaluate the associations of clinical, and radiological features with relapse. A *P* value < 0.05 indicated statistical significance for all analyses. All statistical analyses were performed with Statistical Package for the Social Sciences software, version 20.0 (SPSS Inc., Chicago, IL, USA).

## Results

**Fig. 3:**
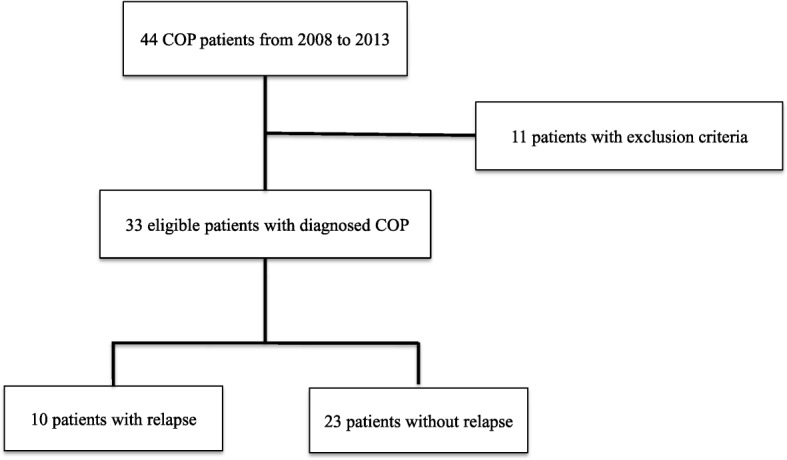
Patient selection flow chart. Of the 33 eligible patients diagnosed as COP, 10 patients were classified to relapse group, and 23 patients were non-relapse

Thirty-three patients fulfilled the selection criteria, of whom 10 (30.3%) relapsed and 23 (69.7%) did not (Fig. 3). The clinical characteristics of both groups are shown in Table 1, and there was no significant difference. The most common symptom in all patients was cough (81.8%), followed by fever (66.6%). One patient in the non-relapse group presented with chest pain. Physical findings included crackles in 60.6% and wheezing in 15.1% of all patients.

**Table 1:** Comparison of clinical characteristics between non-relapse and relapse groups at initial episode in univariate analysis

Variable	Non-relapse group (*n* = 23)	Relapse group (*n* = 10)	P
Age ± SD	74 ± 14	71 ± 10	NS
≥70 years	8 (35)	2(20)	NS
Male	14 (61)	4 (40)	NS
Female	9 (39)	6 (60)	NS
Smoking history			
Never smoked	12 (52)	8 (80)	NS
Prior smoker	8 (35)	2 (20)	NS
Current smoker	3 (13)	1 (10)	NS
Cough	19 (83)	8 (80)	NS
Sputum	13 (57)	1 (10)	NS
Dyspnea	14 (61)	6 (60)	NS
Fever	15 (65)	7 (70)	NS
Chest pain	1 (4)	0 (0)	NS
Crackles	15 (65)	5 (50)	NS
Wheezing	4 (17)	1 (10)	NS

Values are presented as No. (%). Abbreviation: *NS* not significant

Four patients were still on steroid treatment (mean prednisone dose 6.8 ± 3.7 mg; range 2.5–12.5 mg/day) when the first relapse occurred. The mean time to first relapse was 476 ± 445 days (range, 70–1682 days). All patients were treated with increased steroid dose when relapse occurred, and the mean prednisone equivalent dose at that time was 24 ± 8 mg/day.

Comparisons of serum parameters (Table 2) revealed no significant differences between groups. Table 3 compared the radiological findings between groups. Chest radiographs revealed consolidation in 30 patients (90.9%), which was bilateral in 20 (60.6%). The proportion of patients with a bilateral shadow pattern was significantly higher in the relapse group (90% vs. only 47.8% in the non-relapse group, *p* = 0.02). Similarly, incidence of traction bronchiectasis was also significantly higher in the relapse group (60% vs. 17.3% in the non-relapse group, p = 0.02). Although there was no significant difference in the lesion pattern distribution between groups (consolidation, ground-glass attenuation, or nodules), the proportion of patients with partial remission after steroid treatment was significantly higher in the relapse group (90% vs. 43.3% in the non-relapse group, *p* = 0.01). Migratory infiltrative shadows were observed in five patients in the entire cohort (15.1%) and did not differ significantly between groups.

**Table 2:** Comparison of serum parameters between non-relapse and relapse groups at initial episode in univariate analysis

Variable (No. without/with relapse)	Non-relapse group	Relapse group	P
Serum parameters			
WBC (23/10), μL	9021 ± 3733	7580 ± 1975	NS
LDH (23/10), IU/L	242 ± 88	235 ± 63	NS
ALP (23/10), IU/L	285 ± 105	321 ± 123	NS
CRP (23/10), mg/dl	10 ± 7	8 ± 7	NS
KL-6 (23/10), U/ml	507 ± 273	440 ± 341	NS
SP-D (23/10), ng/ml	120 ± 80	120 ± 41	NS

Values are presented as mean ± SD. Abbreviations: *WBC* white blood cell, *LDH* lactate dehydrogenase, *ALP* alkaline phosphatase, *CRP* C reactive protein, *KL-6* Krebs von den Lungen-6, *SP-D* surfactant protein, *NS* not significant. *P* value was not significant for any variable

**Table 3:** Comparison of chest CT scan findings between non-relapse and relapse groups at initial episode in univariate analysis

	Non-relapse group (*n* = 23)	Relapse group (*n* = 10)	P
Pattern of lesion			
Consolidation	21 (91)	9 (90)	NS
Ground-glass opacity	19 (83)	8 (80)	NS
Nodules	4 (17)	2 (20)	NS
Bilateral	11 (48)	9 (90)	0.02
Pleural effusion	2 (9)	0 (0)	NS
Traction bronchiectasis	4 (17)	6 (60)	0.02
Location of lesion			
RUL	18 (78)	6 (60)	NS
RML	10 (43)	4 (40)	NS
RLL	19 (83)	7 (70)	NS
LUL	12 (52)	5 (50)	NS
LLL	11 (48)	6 (60)	NS
Reaction to steroid treatment on chest CT			
Partial remission	10 (43)	9 (90)	0.01

Values are presented as No. (%). Abbreviations: *RUL* right upper lobe, *RML* right middle lobe, *RLL* right lower lobe, *LUL* left upper lobe, *LLL* left lower lobe, *NS* not significant

There was no significant difference in the mean period of steroid treatment or cumulative equivalent dose of prednisone between groups (Table 4). All patients underwent antibiotic therapy before starting the steroid treatment. Eleven patients with severe respiratory disorder at onset received high-dose pulse intravenous steroid treatment with 1 g methylprednisolone for 3 days (30.4% in the non-relapse group vs. 40% in the relapse group, NS).

**Table 4:** Comparison of steroid treatment between non-relapse and relapse groups at initial episode in univariate analysis

Variable	Non-relapse group (*n* = 23)	Relapse group (*n* = 10)	P
Cumulative dose of prednisone, mg/kg	79 ± 47	93 ± 51	NS
Average time of steroid treatment, days	282 ± 163	281 ± 174	NS

Values are presented as mean ± SD. *P* value was not significant for any variable. Abbreviation; *NS* not significant

Thus, the relapse group included a significantly higher proportion of patients with bilateral shadow pattern, traction bronchiectasis, and partial remission as indicated by univariate analysis. However, there were no significant differences by multivariate Cox regression analysis.

## Discussion

This result that radiographic findings may be more important factor than clinical findings, and therapeutic passage is worth investigating further in the large-scale trials. As a result, we identified the three radiographic features, such as bilateral shadow pattern, traction bronchiectasis, and signs of partial remission (residual shadow), may have possibility the predictive factors of relapse. Four of the 10 patients (40%) in the relapse group exhibited all these three radiographic factors, far higher than that in the non-relapse group (8.7%), and three of the four patients had multiple relapses. Okada et al. [14] reported that CT findings of traction bronchiectasis and architectural distortion in COP patients were associated with serum KL-6 levels. In the present study, however, there was no significant difference in serum KL-6 between groups, but 3 of the 10 relapse patients had both serum KL-6 > 500 U/ml and traction bronchiectasis on chest images.

As regards steroid treatment of COP, there was no significant difference in the mean period of steroid treatment or cumulative equivalent dose between groups. There was a possibility that some patients with COP might have unnecessary and excessive steroid treatment. We need to comprehensively analyze clinical, and radiological findings to avoid the risk of side effects by unnecessary steroid treatment, and we should decide therapeutic dose and treatment period of steroid for each patient.

The present study has some limitations. First, the period of treatment and the timing of steroid tapering were not unified because of retrospective study. Although we statistically found no difference in the duration of steroid treatment or cumulative dose of prednisone, we do occasionally encounter COP relapse during tapering or after steroid treatment is stopped. In addition to the guidelines of steroid treatment proposed by Epler [3] and Schwarz and King [4], Lazor et al. [12] proposed the following standardized GERM“O”P protocol: 0.75 mg/kg/day prednisone for 4 weeks, 0.5 mg/kg/day for 4 weeks, 20 mg/day for 4 weeks, 10 mg/day for 6 weeks, and finally 5 mg/day for 6 weeks. Using GERM“O”P, patients received a significantly lower accumulated corticosteroid dose without higher relapse rate, morbidity, or mortality. It was also reported that treatment of first relapse with high-dose corticosteroids was associated with a higher rate of treatment side effects without objective benefit. Although severe adverse events due to steroid treatment were not observed in the present study, we expect that protocol as describes above will be proposed in the future by large prospective clinical trials. Second, we could not evaluate the results of pulmonary function tests such as lung volume and diffusion capacity for carbon monoxide because of the lack of available records for review. Third, the number of registered patients in the present study was small because this was conducted in one hospital. Despite the above limitations, we believe this study has valuable implications for clinical practice.

## Conclusion

We identified several clinical factors that differed significantly in frequency between relapse and non-relapse COP patients. Although there were no significant differences by multivariate Cox regression analysis, we suggest that chest CT scans at initial episode are strong candidate predictive factors for relapse. Identification of such factors could be used to guide preventative treatments, thereby preventing patient distress, avoiding unnecessary steroid treatment, and possibly improving long-term prognosis. We hope that the recurrence factors will be clarified by a long-term study in the future.

## Abbreviations

AFOP: Acute fibrinous organizing pneumonia
ALP: Alkaline phosphatase
COP: Cryptogenic organizing pneumonia
CT: Computerized tomography
KL-6: Krebs von den Lungen-6
LDH: Lactate dehydrogenase
SP-D: Surfactant protein D
WBC: White blood cell
WBC: White blood cell
